# Clinical findings and risk factors for clinical outcomes in dogs with myxomatous mitral valve disease hospitalized for cardiogenic pulmonary edema

**DOI:** 10.3389/fvets.2026.1749038

**Published:** 2026-05-08

**Authors:** Chung-Yao Yin, Ta-Li Lu, John Rush, Tung-Ju Lee, Chao-Yi Chang

**Affiliations:** 1 Chuan Animal Hospital, Taipei, Taiwan; 2 Tufts University Cummings School of Veterinary Medicine, North Grafton, MA, United States; 3 American College of Veterinary Internal Medicine, Greenwood Village, CO, United States

**Keywords:** cardiogenic pulmonary edema (CPE), congestive heart failure (CHF), dog, dyspnea, myxomatous mitral valve disease, outcomes, risk factors, Tufts Dyspnea Score

## Abstract

**Background:**

Cardiogenic pulmonary edema (CPE) is a serious development for dogs with myxomatous mitral valve disease (MMVD). Due to the hemodynamic impact of MMVD, and changes in left atrial pressure and pulmonary capillary pressure, CPE commonly requires hospitalization.

**Hypothesis/Objectives:**

To characterize clinical findings particularly in respiratory rate and breathing effort in dogs with MMVD hospitalized with CPE and assess them as risk factors focusing on selected clinical outcomes.

**Animals:**

100 dogs with MMVD hospitalized with CPE were included, with a median age of 12.0 years and a median body weight of 3.31 kg. Fifty-five dogs were male and 45 were female.

**Methods:**

Prospective cohort study measuring clinical parameters and Tufts Dyspnea Score (TDS) during hospitalization to assess outcomes. Descriptive statistics and regression analysis were used.

**Results:**

Upon hospital presentation, the median respiratory rate was 64 breaths per minute, the median TDS was 5 (ranging from 0 to 10). The median duration of oxygen administration was 20 h. Ninety-five of 100 dogs survived to hospital discharge. For the 93 dogs with follow-up information, 17 experienced cardiac death and 22 experienced re-hospitalization for CPE within 2 months post-discharge. Lower minimum TDS within 12 h was found to be associated with survival to hospital discharge. The administration of parenteral inotropes and higher minimum respiratory rate within 12 h were associated with cardiac death within 2 months post-discharge. Dogs with a louder murmur at presentation were more likely to experience re-hospitalization due to CPE within 2 months post-discharge.

**Conclusions and clinical importance:**

We observed a high survival rate to discharge in dogs with MMVD hospitalized with CPE; however, cardiac death and rehospitalization due to CPE within 2 months post-discharge were common in these dogs. No clinical parameter measured before initiating treatment was associated with in-hospital mortality. However, risk factors such as systolic blood pressure below 90 mmHg upon hospital presentation, administered parenteral inotropes and the minimum respiratory rate during the initial 12 h have been associated with an increased risk of cardiac death within 2 months post-discharge.

## Introduction

Myxomatous mitral valve disease (MMVD) stands as the predominant heart ailment among dogs across numerous regions globally (1). Cardiogenic pulmonary edema (CPE) is a serious development for dogs with MMVD as they progress to congestive heart failure, often necessitating hospitalization for its management. In human heart failure patients, both those managed in hospital and as out patients, validated multivariable risk scores are valuable for estimating the risk of mortality (2). In previous research within the field of human medicine, the in-hospital mortality rate for hospitalized heart failure patients was observed between 3 and 9% (3–5). Azotemia, hypotension, hyponatremia, age, tachypnea, tachycardia, and the presence of comorbidities have been linked to heightened mortality rates in humans admitted to the hospital due to heart failure (3–5).

In veterinary medicine, previous retrospective studies indicated that dogs experiencing acute congestive heart failure (CHF) and developing hypokalemia exhibited a more favorable likelihood of surviving to hospital discharge (6). Conversely, dogs presenting with hyponatremia or hyperglycemia had a less favorable prognosis in another study (7). Hypochloremia has also been identified as an important predictor of the stage of heart disease and the oral furosemide dose in CHF dogs (8–11). A previous study focusing on dogs with stable ACVIM Stage C MMVD, the normalized left ventricle internal diameter at the end of diastole was also identified as an independent predictor for the first recurrence of congestive signs within 180 days (12).

Monitoring breathing status, often assessed through measures like respiratory rate, serves as a common indicator for evaluating control of left-sided CHF and subclinical left-sided heart disease in both dogs and cats (13, 14). Additionally, assessment of respiratory rate aids in predicting the onset of CHF (15, 16). Nevertheless, the effectiveness of utilizing respiratory rate or breathing effort to monitor hospitalized patients with CPE and predict their outcomes remains uncertain.

The aim of our study was to characterize respiratory rate and breathing effort in dogs with MMVD hospitalized for CPE and assess them as risk factors focusing on selected clinical outcomes such as duration of oxygen administration, in-hospital mortality, cardiac-related deaths within 2 months post-discharge, and rehospitalization due to CPE within 2 months post-discharge. Our hypothesis was that among dogs with MMVD hospitalized for CPE, faster improvement in respiratory rate or breathing effort would be linked to better outcomes.

## Materials and methods

### Study design

In this prospective cohort study, dogs were eligible for inclusion between June 2020 and November 2022 at a single, referral private hospital. Dogs were prospectively enrolled on a consecutive basis during the study period. The sample size was determined by the total number of eligible cases presenting during the study period. The study’s inclusion criteria encompassed client-owned dogs aged over 5 years, weighing less than 20 kilograms, diagnosed with MMVD Stage C (1), currently experiencing CPE, and having received a medical assessment indicating the need for hospitalization due to CPE. All owners consented to the data collection during the hospitalization period.

### Inclusion and exclusion criteria

The diagnosis of MMVD involved a combination of characteristic left apical regurgitant quality systolic murmur and the presence of echocardiographic observations (1). These observations included mitral valve leaflet thickening and subjective left atrial enlargement assessed through 2D echocardiography, and identification of mitral valve regurgitation via color Doppler examination.^1^ The diagnosis of CPE relied on the following criteria: the presence of clinical signs consistent with CPE (such as tachypnea, restlessness, respiratory distress, or cough), and radiographic evidence of pulmonary edema. A modified composite CHF score from thoracic radiographs was employed, and dogs with a score greater than 4 were included (15). Dogs were excluded if they had other concurrent cardiac defects like cardiomyopathy or congenital heart disease. Likewise, dogs with pulmonary hypertension unrelated to left-sided heart disease, or those concurrently affected by pulmonary parenchymal conditions like bacterial pneumonia or noncardiogenic pulmonary edema, were not considered. Dogs afflicted with other severe systemic diseases (e.g., diabetes mellitus, malignant neoplasia, International Renal Interest Society Stage 4 chronic kidney disease, status epilepticus and acute pancreatitis) also were excluded from the study.

### Initial data collection

Data were collected both upon the initial presentation to the hospital and throughout the period of hospitalization. Data recorded upon hospital presentation included signalment, body weight, chief complaints, medical history and physical examination findings. These findings included heart rate, systolic blood pressure, mucous membrane color, grade of murmur, the presence or absence of crackles, respiratory rate and Tufts Dyspnea Score (TDS). Information regarding systolic blood pressure was gathered using either a high-definition oscillometric (HDO) blood pressure monitor or a conventional Doppler blood pressure machine.^2^^,^^3^

### Tufts Dyspnea Score

TDS is a scale intended to categorize the severity of respiratory distress in dogs and cats with cardiopulmonary disease. TDS is ranging from 0 to 10, where 0 indicates no dyspnea, 1–3 indicates mild dyspnea, 4–6 indicates moderate dyspnea, and 7–10 indicates severe dyspnea. Before starting data collection, we compiled videos of dogs exhibiting varying severities of dyspnea and shared these with all clinicians involved in scoring, aiming for consistent evaluations across the team. As noted in Supplementary Appendix 1, the criteria to assess columns should be used as a guideline (e.g., many individuals with this score have this parameter), rather than an absolute criteria for scoring. Inter-observer variability was assessed using 30 videos of dogs with varying degrees of dyspnea, which were independently scored by four veterinarians using the TDS. To assess intra-observer variability, eight videos were randomly repeated within the survey.

### Imaging protocols

Thoracic radiograph was performed in a dorsoventral view for each dog at presentation to the hospital. The data collected included the modified composite CHF score, the pattern and distribution of pulmonary infiltrates and the Modified Murray Lung Injury Score (MMLIS) as outlined in previous studies (17–19). Radiographic exams were completed within 2 hours of hospitalization for dyspneic dogs unable to undergo the exams at presentation. Echocardiographic assessments were conducted with an ultrasound unit (see text footnote 1) at different timepoints throughout the hospital stay.

### Oxygen supplementation

These critical dogs started oxygen administration before radiographic examinations. Eventually, all dogs were transferred to an intensive care unit (ICU) cage,^4^ which is furnished with oxygen supplementation and allowed control of humidity and temperature. The administration of oxygen would commence at 40% concentration, and adjustments to the oxygen concentrations made in a gradual manner based on the patient’s respiratory status. An oxygen mask was used only temporarily when dogs needed to be removed from the cage for procedures such as blood withdrawal, blood pressure measurement, or radiographic examinations.

### Monitoring and treatment

Throughout the hospitalization, the respiratory rate and TDS were assessed on an hourly basis. Respiratory rate and TDS during the first 12 h of hospitalization was gathered to compute the minimum, median, and maximum values. Additionally, at the 12-h time point of the hospitalization period, body weight, heart rate, and systolic blood pressure were re-evaluated. Within the timeframe of 6 to 24 h after admission, a subsequent dorsoventral radiograph was performed, and the MMLIS was re-calculated. Both the actual numerical value and the percentage change of MMLIS between the initial presentation and the follow-up were calculated. Data pertaining to the cumulative parenteral furosemide dose administered during the first 12 and 24 h of hospitalization, as well as the administration of parenteral inotropes like dobutamine and milrinone, was documented.

### Outcomes and follow-up

Four outcome variables were assessed in this study. First, duration of oxygen administration was treated as a continuous variable, representing the number of hours a patient received oxygen while hospitalized. Second, survival to hospital discharge was treated as a binary variable, categorizing patients who did not survive to hospital discharge, either due to natural cardiac fatalities or euthanasia, as non-survivors. Third, cardiac death occurring within 2 months post-discharge was evaluated. The number of days to cardiac death was treated as a continuous variable. Cardiac deaths were confirmed through direct contact with the owner at the 2-month after discharge. Lastly, excluding those with cardiac deaths within 2 months post-discharge, we investigated whether patients experienced rehospitalization due to CPE during that period. The number of days to rehospitalization for CPE was documented based on medical records and treated as a continuous variable.

### Statistical analysis

All statistical tests were performed with commercial statistical software.^5^ Continuous data are expressed as median and IQR. Wilcoxon rank sum tests were employed to compare variables between the time of presentation and the subsequent follow-up assessment. Linear regression was employed to evaluate the relationship between different variables and the duration of oxygen administration during hospitalization. Variables that were significant (*p* < 0.05) in linear regression analysis were entered into multivariable stepwise selection multiple regression analysis. Logistic regression analysis was used to assess the association of variables with survival to hospital discharge. Variables that were significant (*p* < 0.05) in univariable logistic regression analysis were entered into multivariable stepwise selection logistic regression analysis. Cox proportional hazards regression analysis was performed to evaluate the relationship between different variables and cardiac death or rehospitalization due to CPE within 2 months after discharge, respectively. Variables that were significant (*p* < 0.05) in univariable Cox proportional hazards regression analysis were entered into multivariable stepwise selection Cox proportional hazards regression analysis. Tests for collinearity between variables were done by calculating variance inflation factor (VIF) using enter selection multiple regression analysis. Variables with VIF > 10 were selectively removed before entering multivariable regression analysis. The intraclass correlation coefficient (ICC) was used to evaluate inter- and intra-observer variability of the TDS.

## Results

### Demographics, chief complaints and medical history

**Figure 1:**
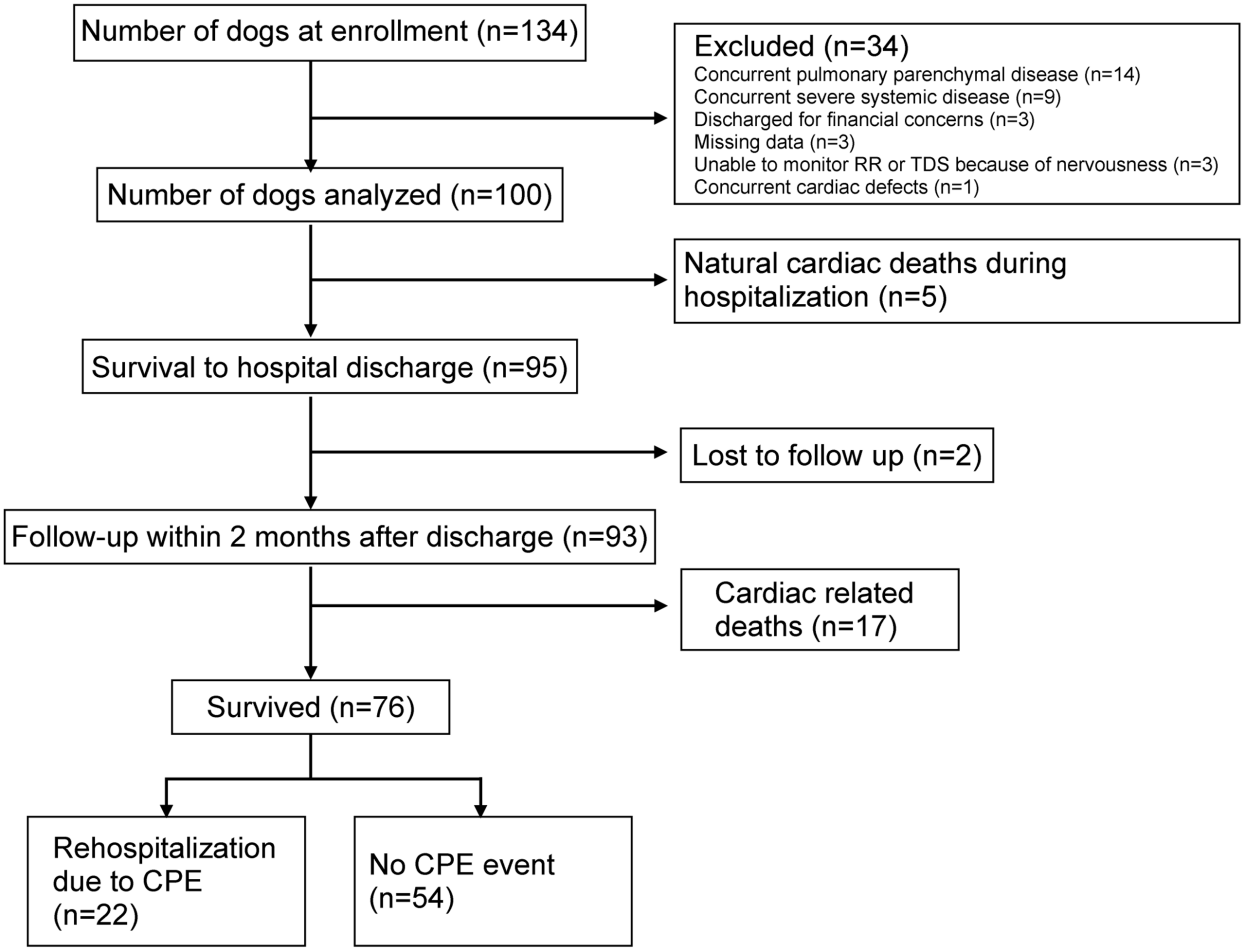
Flow chart of dog enrollment and outcome results. RR, respiratory rate; TDS, Tufts Dyspnea Score; CPE, cardiogenic pulmonary edema.

One hundred thirty-four dogs were enrolled in this study. Thirty-four dogs were excluded from the study: 14 had concurrent pulmonary parenchymal disease, 9 had severe systemic diseases, 3 were discharged due to financial concerns, 3 had significant missing data, 3 were too nervous to allow monitoring of respiratory rate or TDS, and 1 had concurrent cardiac defects. 100 dogs were eligible for final analysis (Figure 1). The median age was 12.0 years (IQR, 11.0–14.0) and a median body weight of 3.31 kg (IQR, 2.64–4.58). Thirty-five dogs were neutered male, 20 were intact male, 38 were neutered female and 7 were intact female. The 100 dogs were comprised of 52 Maltese, 13 Chihuahua, 9 Pomeranian, 8 Miniature schnauzer, 7 Miniature poodle, 2 Spitz, 2 Yorkshire terriers, 1 mixed breed dog, and one dog each of Cavalier King Charles Spaniel, Dachshund, Fox terrier, Shetland Sheepdog, Shiba Inu, and Shih Tzu.

Prevalent chief complaints encompassed tachypnea (81/100, 81%) and increased respiratory effort (76/100, 76%). Additional presenting complaints included coughing (47/100, 47%), anorexia (27/100, 27%), lethargy (26/100, 26%), collapse (14/100, 14%) and hemoptysis (6/100, 6%). Fifty-seven dogs were diagnosed with first-onset CPE. Forty-three dogs had a previous history of CPE, the majority of these cases with only one prior episode (25/43, 58%), while 5 dogs (5/43, 12%) had two prior episodes of CPE and 13 (13/43, 30%) with three or more episodes of CPE. Upon hospital presentation, 74 dogs were already receiving cardiac-related medication (Table 1), with nearly all of them having been prescribed pimobendan (73/74, 99%). Preexisting, concurrent diseases were present in 43 dogs. The most common concurrent diseases were chronic kidney disease (17/100, 17%), pulmonary hypertension (11/100, 11%), benign neoplasia (6/100, 6%), tracheal collapse (5/100, 5%), chronic pancreatitis (3/100, 3%) and anemia (3/100, 3%). Within the week prior to hospital admission for CPE, 12 dogs had a history of previous grooming, 4 had undergone prior systemic glucocorticoid therapy to control coughing, and 1 had received parenteral fluid therapy.

**Table 1:** List of medications administered before presentation to the hospital for onset of acute CPE.

Drugs	Number of dogs	Median dose (range) (mg/kg/day)
Pimobendan	73	0.7 (0.3–5.1)
Furosemide	42	2.0 (0.2–6.0)
Torsemide	16	0.3 (0.1–1.6)
Spironolactone	49	2.0 (0.9–9.7)
ACEI	32	1.0 (0.2–1.0)
Sildenafil	14	2.1 (1.0–6.6)
Amlodipine	9	0.2 (0.1–0.4)

ACEI, angiotensin-converting enzyme inhibitors. ACEI included enalapril and benazepril.

### Physical examination and thoracic radiograph

On physical examination, the median respiratory rate upon presentation was 64 breaths per minute (IQR, 52–84), while the median TDS was 5 (IQR, 3–6). Nine dogs had a respiratory rate less than 40 breaths per minute at the presentation, with 7 out of 9 (78%) exhibited at least mild dyspnea based on TDS assessment (TDS of 2 in 2 dogs, 3 in 6 dogs, 8 in one dog). The median heart rate upon presentation was 150 beats per minute (IQR, 120–169), and the median systolic blood pressure was 118 mmHg (IQR, 104–131). A murmur intensity greater than or equal to three out of six was identified in every dog. Among them, 2 dogs had a grade III/VI murmur, 48 had a grade IV/VI murmur, 45 had a grade V/VI murmur, and 5 had a grade VI/VI murmur. In addition to murmur, pulmonary crackles were discernible in 77 dogs, with the majority exhibiting bilateral crackles (49/77, 64%), while the remaining had unilateral crackles (28/77, 36%). Mucous membrane color abnormalities, either pallor or cyanosis, were observed in 59 dogs (59/100, 59%).

Thoracic radiographs exhibited infiltrates compatible with CPE in all dogs. The median MMLIS upon presentation was 4 (IQR, 2–6). An unstructured interstitial pattern was recognized in 45 dogs (45/100, 45%), 44 (44/100, 44%) displaying a lung pattern that combined both unstructured interstitial and alveolar patterns, whereas only 11 dogs (11/100, 11%) demonstrated an exclusive alveolar pattern.

### Treatment and outcome

Every dog included in this study received oral pimobendan and parenteral furosemide during hospitalization. Among these, 92% of the dogs (92/100) were managed using a furosemide constant rate infusion (CRI), while the remaining 8% (8/100) received treatment solely through intermittent furosemide bolus administration. The median cumulative parenteral furosemide dose administered to all dogs in the initial 12-h period was 18.7 mg/kg (IQR, 13.6–24.1), while over the initial 24-h span, it amounted to 25.3 mg/kg (IQR, 17.1–36.1). Parenteral inotropes were administered to 19 dogs (19/100, 19%) during their hospitalization, among which 16 were treated using dobutamine, and 3 received milrinone. All dogs in this study received oxygen supplementation, and they were all initially hospitalized in the ICU cage with supplemental oxygen.

**Figure 2:**
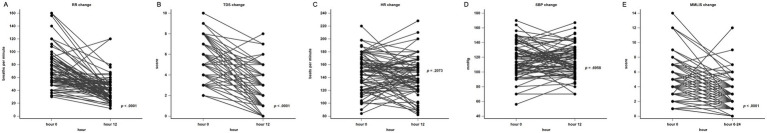
Change plots showing the difference in clinical parameters from the initial presentation to the initial 12 hours of hospitalization. **(A)** changes in respiratory rate (RR); **(B)** changes in Tufts Dyspnea Score (TDS); **(C)** changes in heart rate (HR); **(D)** changes in systolic blood pressure (SBP); **(E)** changes in the Modified Murray Lung Injury Score (MMLIS) through radiographs.

After 12 h of hospitalization the median respiratory rate decreased from 64 breaths per minute (IQR, 52–84) to 36 breaths per minute (IQR, 28–48) (*p* < 0.001), while the median TDS decreased from 5 (IQR, 3–6) to 2 (IQR, 1–3) (*p* < 0.001). Additionally, the initial median heart rate was 150 beats per minute at baseline (IQR, 120–169) and the median heart rate at 12 h post hospitalization was 144 beats per minute (IQR, 126–160) (*p* = 0.21). The initial median systolic blood pressure was 118 mmHg (IQR, 104–131) and the median systolic blood pressure at 12 h post hospitalization was 110 mmHg (IQR, 100–128) (*p* = 0.7). In the follow-up radiograph conducted within 6–24 h of hospitalization, the median MMLIS decreased from 4 (IQR, 2–6) to 2 (IQR, 1–4) (*p* < 0.001) (Figure 2).

Dogs were hospitalized for a median period of 34 h (IQR, 23–52), and the median duration of supplemental oxygen administration was 20 h (IQR, 12–32). A total of 63% (63/100) of the dogs underwent oxygen administration for less than 1 day, 24 out of 100 (24%) stayed in oxygen for 1 to 2 days, 7 out of 100 (7%) remained in oxygen for 2 to 3 days, and 6 out of 100 (6%) stayed beyond 3 days.

In terms of survival probability, 95 out of 100 dogs (95%) successfully survived to hospital discharge (Figure 1). Among the five non-surviving dogs, natural deaths occurred due to cardiac disease while hospitalized. All of them experienced their demise within the first 24 h of hospitalization. No dogs were euthanized. Apart from these five cases, in four dogs, symptoms of CPE could not be fully resolved despite aggressive treatment, indicating that 91 out of 100 dogs (91%) were free of overt clinical signs of CPE at the time of hospital discharge.

Out of the 93 dogs with follow-up data, 17 dogs (17/93, 18%) experienced cardiac death (15 due to natural cardiac disease-related deaths and 2 euthanized due to frequent relapse of CPE) within 2 months following discharge. Excluding those that suffered cardiac death, 22 dogs (22/76, 29%) were rehospitalized due to CPE within 2 months after discharge (Figure 1).

Blood tests were done based on the veterinarian’s judgment of the patient’s condition. While not part of the study protocol, serum kidney values and electrolyte data were obtained from 98 dogs, with measurements taken at various time points during hospitalization. Median serum urea nitrogen was 48 mg/dL (IQR 36–66), median serum creatinine was 1.5 mg/dL (IQR 1.0–1.9), median serum sodium was 143 mmol/L (IQR 139–147), median serum potassium was 3.8 mmol/L (IQR 3.4–4.1), median serum chloride was 105 mmol/L (IQR 101–108). Serial measures of kidney function at specific time points were not part of the study design; however, during hospitalization, 28 dogs (28/98, 29%) were documented to have developed a serum creatinine level above the reference range (0.5–1.8 mg/dL) for the laboratory.

### Prognostic risk factors

In the univariable analysis aimed at assessing the duration of oxygen administration, several factors exhibited positive associations (see footnotes in Table 2) and were subsequently entered in the multivariable stepwise selection multiple regression analysis. In the multiple regression analysis, several factors showed associations with longer oxygen administration, including: a higher cumulative parenteral furosemide dose during the 12–24 h period (*p* < 0.001, Coef = 1.1631, 95% CI = 0.6857–1.6405), the administration of parenteral inotropes (*p* < 0.001, Coef = 23.4152, 95% CI = 13.7918–33.0385), a higher pre-hospitalization dose of furosemide (*p* = 0.0011, Coef = 3.7487, 95% CI = 1.5476–5.9498) and a higher MMLIS upon follow up thoracic radiographs (*p* = 0.019, Coef = 2.5388, 95% CI = 0.4273–4.6503) (Table 2).

**Table 2:** Variables associated with the duration of oxygen administration (hours) identified in the multiple linear regression analysis.

Variable	*p*-value	Coef (95% CI)	Std. error	*t*	VIF
Total furosemide dose during 12–24 h (mg/kg)	<0.0001	1.1631 (0.6857–1.6405)	0.2402	4.8426	1.926
Administration of parenteral inotropes (y/n)	<0.0001	23.4152 (13.7918–33.0385)	4.8417	4.8362	1.383
Pre-hospitalization dose of furosemide (mg/kg)	0.0011	3.7487 (1.5476–5.9498)	1.1074	3.3852	1.012
MMLIS upon follow up	0.019	2.5388 (0.4273–4.6503)	1.0623	2.3899	2.055

MMLIS, Modified Murray Lung Injury Score; TDS, Tufts Dyspnea Score. Variables that exhibited positive associations in univariable linear regression analysis were included in the multivariable stepwise selection multiple regression analysis, which identified the following factors: higher cumulative parenteral furosemide dose during the first 12 h (*p* < 0.001) and the 12–24 h period (*p* < 0.001); higher MMLIS upon follow-up thoracic radiographs (*p* < 0.001); administration of parenteral inotropes (*p* < 0.001); longer time required for respiratory rate to drop below 40 breaths per minute (*p* = 0.03); higher median respiratory rate during the first 12 h (*p* = 0.04); elevated TDS at presentation (*p* = 0.02); and higher minimum (*p* < 0.001), median (*p* < 0.001), and maximum (*p* = 0.01) TDS during the first 12 h. Additionally, longer time for TDS to drop below 3 (*p* = 0.001), as well as higher pre-hospitalization doses of furosemide (*p* = 0.01) and torsemide (*p* = 0.02) were also noted. Example of the interpretation: dogs administered with parenteral inotropes had an estimated 23-h longer duration of oxygen administration compared to dogs that did not receive inotropes.

In the univariable analysis aimed at assessing survival to hospital discharge, several factors exhibited positive associations (see footnotes in Table 3) and were subsequently entered in the multivariable stepwise selection logistic regression analysis. In the multivariable logistic regression, each one-unit increase in minimum TDS during the initial 12 h was associated with an 82% decrease in the odds of a dog surviving to hospital discharge (*p* = 0.003, OR = 0.18, 95% CI = 0.06–0.56) (Table 3). In the univariable analysis aimed at assessing cardiac death within 2 months after discharge, several factors exhibited positive associations (see footnotes in Table 4) and were subsequently entered in the multivariable stepwise selection Cox proportional hazards analysis. In the multivariable Cox proportional hazards analysis, dogs presenting with systolic blood pressure below 90 mmHg upon hospital presentation had 6.3 times the hazard of cardiac death within 2 months post-discharge (*p* = 0.02, HR = 6.3, 95% CI = 1.3–30) and those administered parenteral inotropes had 5.3 times the hazard of cardiac death within 2 months post-discharge (*p* = 0.025, HR = 5.3, 95% CI = 1.2–22) compared with dogs without these risk factors. Each one-unit increase in minimum respiratory rate during the initial 12 h was associated with a 10% increase in the hazard of cardiac death within 2 months post-discharge (*p* = 0.003, HR = 1.1, 95% CI = 1.0–1.2) (Table 4).

**Table 3:** Variables associated with survival to hospital discharge identified in the multivariable logistic regression analysis.

Variable	Coef	Std. error	*p*-value	Odds ratio (95% CI)
Minimum TDS during 12 h	−1.6896	0.5685	0.003	0.18 (0.06–0.56)

TDS, Tufts Dyspnea Score.

Variables that exhibited positive associations in the univariable logistic regression analysis were included in the multivariable stepwise selection logistic regression analysis. The following factors were identified: presence of bilateral pulmonary crackles at presentation (*p* = 0.01), higher minimum (*p* < 0.001), median (*p* = 0.003), and maximum (*p* = 0.004) respiratory rates during the first 12 h, as well as higher minimum (*p* < 0.001), median (*p* = 0.003), and maximum (*p* = 0.01) TDS during the initial 12 h. Example of the interpretation: Each one-unit increase in minimum TDS during the initial 12 h of hospitalization was associated with an 82% decrease in the odds of a dog surviving to hospital discharge.

**Table 4:** Variables associated with cardiac deaths within 2 months post-discharge identified in the multivariable Cox proportional hazards analysis.

Variable	Coef	Std. error	*p*-value	Hazards ratio (95% CI)
SBP < 90 mmHg at hospital presentation (y/n)	1.8395	0.7928	0.02	6.3 (1.3–30)
Administration of parenteral inotropes (y/n)	1.6725	0.7434	0.025	5.3 (1.2–22)
Minimum RR within 12 h (bpm)	0.0892	0.0295	0.003	1.1 (1.0–1.2)

RR, Respiratory rate; bpm, breaths per minutes; SBP, systolic blood pressure. Variables that exhibited positive associations in the univariable Cox proportional hazards analysis were included in the multivariable stepwise selection Cox proportional hazards analysis. The following factors were higher cumulative parenteral furosemide dose within the first 24 h (*p* = 0.03); higher MMLIS at hospital presentation (*p* = 0.02) and at follow-up (*p* = 0.003); administration of parenteral inotropes (*p* = 0.0008); higher minimum (*p* = 0.0005) and median (*p* = 0.01) respiratory rate during the first 12 h; systolic blood pressure below 90 mmHg at hospital presentation (*p* = 0.002); longer time for TDS to drop below 3 (*p* = 0.007); and higher minimum (*p* = 0.0002), median (*p* = 0.006), and maximum (*p* = 0.01) TDS during the initial 12 h. Example of the interpretation: Dogs administered parenteral inotropes had 5.3 times the hazard of cardiac death within 2 months post-discharge compared to dogs that did not receive parenteral inotropes.

In the univariable analysis aimed at assessing rehospitalization due to CPE within 2 months after discharge, several factors exhibited positive associations (see footnotes in Table 5) and were subsequently entered in the multivariable stepwise selection Cox proportional hazards analysis. In the multivariable Cox regression analysis, each one-unit increase in the grade of murmur was associated with a 6.5 times increase in the hazard of rehospitalization due to CPE within 2 months post-discharge (*p* = 0.0001, HR = 6.5, 95% CI = 2.5–17) (Table 5).

**Table 5:** Variables associated with rehospitalization due to CPE within 2 months post-discharge identified in the multivariable Cox proportional hazards analysis.

Variable	Coef	Std. error	*p*-value	Hazard ratio (95% CI)
Grade of murmur	1.8677	0.4920	0.0001	6.5 (2.5–17)

Variables that exhibited positive associations in the univariable Cox proportional hazards analysis were included in the multivariable stepwise selection Cox proportional hazards analysis. The following factors were higher cumulative parenteral furosemide dose during the 12–24 h period (*p* = 0.05), higher grade of murmur (*p* = 0.0001) and higher pre-hospitalization dose of torsemide (*p* = 0.01). Example of the interpretation: Each one-unit increase in the grade of murmur was associated with a 6.5 times increase in the hazard of rehospitalization due to CPE within 2 months post-discharge.

The number of prior CPE episodes was not associated with the duration of oxygen administration (*p* = 0.2), survival to hospital discharge (*p* = 0.05), cardiac death within 2 months after discharge (*p* = 0.66) or rehospitalization within 2 months after discharge (*p* = 0.09).

The results of inter-observer and intra-observer variability demonstrated very good agreement, with an inter-observer intraclass correlation coefficient (ICC) of 0.9635 (95% CI: 0.9229–0.9826). The intra-observer ICC values for the four observers were 1.000 (95% CI: 1.000–1.000), 0.9951 (95% CI: 0.9754–0.9990), 0.9937 (95% CI: 0.9700–0.9987), and 0.9598 (95% CI: 0.8161–0.9918), respectively.

## Discussion

The results of the present study suggest that dogs with CPE related to MMVD had a high likelihood of survival to hospital discharge, with 95% of dogs being discharged from the hospital. Upon hospital presentation, no clinical parameter measured before initiating treatment was associated with in-hospital survival. However, parameters recorded during the initial 12 h of hospitalization, specifically the minimum TDS during the initial 12 h and the minimum respiratory rate during the initial 12 h, were associated with survival to hospital discharge and cardiac death within 2 months after discharge, respectively.

The predominant chief complaints were primarily associated with respiratory abnormalities, including tachypnea and increased respiratory effort, with 95% of dogs exhibiting such signs. Prior studies on dogs with acute CHF reported respiratory abnormalities in 39 to 100% of cases (6–8). Notably, coughing was observed in 47% of the dogs in our study, mirroring findings from a prior retrospective study on acute CHF in dogs and cats (19). Half of the dogs in our study experienced their first episode of CPE. However, the number of prior CPE episodes was not found to be associated with the duration of oxygen administration, survival to hospital discharge, cardiac death within 2 months after discharge and rehospitalization within 2 months after discharge. A link between prior CHF episodes and worse outcomes may exist, but our small sample size and reliance on owner-reported data from other clinics could have limited detection and introduced inaccuracies.

The median respiratory rate upon admission was 64 breaths per minute, which closely aligns with the prior study findings, where they were reported as 56 to 60 breaths per minute (6, 20). Nine dogs present with a relative normal respiratory rate, but seven dogs exhibited at least mild dyspnea based on TDS assessment with 7 of 9 dogs having TDS ≥ 3. Some dyspneic dogs may present with a relatively normal respiratory rate but an elevated TDS. We consider TDS and RR to be distinct metrics, and both may serve as sensitive parameters for evaluating patients with CPE. As expected, it was observed that all dogs had a murmur intensity ≥3/6, similar to a previous study (21), where no dogs with CHF had a murmur intensity less than 3/6.

Statistically significant reductions in respiratory rate, TDS, and MMLIS were observed during the initial 12 h of treatment. These findings suggest that the majority of dogs were responsive to treatment, with gradual improvement in the severity of CPE. The median duration of hospitalization and oxygen supplementation in our study closely mirrored the findings reported in previous research (8, 20).

The in-hospital mortality rate in our study was similar to that in people with CHF which was between 3 and 9% (3–5). Some recent veterinary studies also had low in-hospital mortality rates, although inclusion criteria between trials can vary widely (8, 19). This contrasts with the higher in-hospital mortality rates observed in earlier veterinary studies, which ranged from 20 to 44% (6, 7). There are several factors that could account for the high survival rate in our study. Firstly, all dogs in our study were small breed dogs (median body weight at presentation 3.31 kg [IQR 2.64–4.58]), with an overrepresentation of Maltese dogs, as noted in previous studies (22, 23). In contrast, other studies on in-hospital mortality included both small and large breed dogs (6, 7). Large breed dogs were determined to have a higher likelihood of systolic dysfunction and arrhythmias such as atrial fibrillation and ventricular arrhythmia (24, 25), factors that could potentially increase mortality rates. A study also revealed that the risk of a negative outcome increases significantly when large breed dogs progress from ACVIM stage B1 to stage B2 or C (26). Secondly, no dogs in our study were euthanized, in contrast to the 73–86% of patients euthanized in previous studies (6, 7). Pet owners’ cultural differences and financial considerations may influence decisions regarding patient care (27). This could potentially explain the variance in euthanasia rates, resulting in a higher survival rate in our study.

In the largest study cohort examining rehospitalization among human patients with heart failure (28), it was reported that 27% of patients were rehospitalized within 30 days. In previous veterinary studies, 26% of dogs experienced a recurrence of CHF or cardiac-related death within 90 days of diagnosis (19), while 24% showed a recurrence of congestive signs within 180 days (12). These results are similar to our findings, where 29% of dogs were rehospitalized for CPE within 2 months after discharge. Therefore, it is crucial to re-emphasize the importance of closely monitoring dogs post-discharge, particularly by paying close attention to resting respiratory rate (13). Frequent recurrences post-discharge remains a challenging issue in the population. Exploring future studies to assess the effectiveness of interventions aimed at preventing these post-discharge recurrences could be highly intriguing.

One study showed that lower urine sodium concentration was associated with longer mean duration of treatment with supplement oxygen in dogs with CHF (29). Even though urinalysis was not conducted in our study, we still found that several variables showed associations with a longer duration of oxygen administration in the multiple regression analysis. We can categorize these variables into two distinct groups. First, there are treatment-related factors, including the administration of parenteral inotropes, higher cumulative parenteral furosemide dose during the 12–24 h period, and the pre-hospitalization dose of furosemide. These parameters may also serve as indicators of a more advanced status of CPE or diuretic resistance. Urine sodium concentration may also play a potential role in assessing diuretic resistance (30). However, this is not the aim of our research, so it cannot be evaluated. Secondly, a parameter that assesses the severity of CPE changes: higher MMLIS upon follow-up thoracic radiographs, which can also be interpreted as reflecting the improvement of CPE severity over time.

Minimum TDS during the initial 12 h appeared to be the only factor with potential relevance for indicating survival probability during hospitalization. However, only five in-hospital deaths occurred, limiting the number of variables that could be reliably included in the logistic regression analysis. Therefore, the results of the multivariable analysis should be interpreted with caution. No clinical parameters at presentation were identified as a predictor of survival to hospital discharge. Hence, we think it is important to consider continuing treatment despite seemingly unfavorable clinical findings at the time of initial presentation, as favorable outcomes may be possible with appropriate treatment and care. Nevertheless, since all fatalities in the current study occurred within the initial 24 h, it is imperative not only to closely monitor the patient’s respiratory status but also recognize that a continuously high TDS during the initial 12 h of hospitalization may signal a deteriorating CPE patient with an elevated risk of mortality. However, we do not support using the minimum TDS during the initial 12 h for recommending euthanasia, as there were patients with higher TDS levels during this period who still survived to hospital discharge.

Administration of parenteral inotropes and a higher minimum RR within 12 h were significantly associated with cardiac death within 2 months after discharge in our study. Parenteral inotropes are most indicated for patients with acute signs of heart failure complicated by hypotension, which potentially explained why a systolic blood pressure of <90 mmHg at hospital presentation was also associated with this outcome. Although dogs treated with injectable inotropes had poorer outcomes, this may reflect more severe underlying cardiac disease in these patients. A higher respiratory rate within the first 12 h of hospitalization further suggests more severe heart failure or treatment failure, which may be due to diuretic resistance. Consequently, even if these patients survive the acute episode, they remain at an increased risk of mortality after discharge.

In our study, the only factor associated with rehospitalization due to CPE within 2 months after discharge was greater murmur intensity at presentation. As noted in previous studies (21), murmur intensity was not only associated with the stage of MMVD and the degree of left atrial dilation, but also with the likelihood of developing CHF or pulmonary hypertension. Although murmur intensity may play a potential role in predicting the recurrence of CHF, it is a sensitive but not specific parameter for assessing the severity of MMVD. For instance, a dog with a mild murmur is unlikely to have severe left atrial enlargement or be in active CHF, whereas a dog with a moderate or severe murmur could present either way. Therefore, this result should be interpreted with great caution.

There were several limitations to this study. All dogs in the study were from a single referral and emergency hospital, primarily consisting of small breeds, particularly Maltese. As a result, the findings may not be fully representative of the general population of dogs with MMVD and CPE. Radiographs were assessed only in a single DV view, as this position is the least stressful for dogs with active CPE. However, the absence of a lateral view radiograph could potentially lead to a misinterpretation of the degree of CPE and an inability to assess parameters like Vertebral Heart Scale (VHS) or Vertebral Left Atrial Size (VLAS). During hospitalization, TDS assessments were conducted by different veterinarians. Our study was unable and not designed to assess the intraobserver and interobserver coefficients of variation for TDS, as there was no record indicating which clinician recorded each TDS. The subjective nature of TDS combined with the lack of data on inter- or intra-observer variation, presents a limitation in using TDS in this study. In addition, the decisions regarding medication doses, frequency and subsequent medication adjustments were made by different veterinarians, which may have impacted the outcomes. Although several studies have indicated that kidney values, serum electrolytes and echocardiographic measurements may be associated with short-term outcomes in both human and veterinary medicine (3–12, 29, 30), we did not request blood tests and echocardiographic measurements for every patient due to practical challenges in a clinical setting. These challenges may arise from the patient’s unstable condition or the owner’s financial considerations. Thus we cannot comment on whether baseline abnormalities or subsequent alterations in kidney values, serum electrolytes and echocardiographic measurements might impact the outcomes which is another limitation of our study. While this study was conducted in a prospective manner, the sample size in our study was relatively small. The limited number of nonsurvivors in our study may have potentially impacted the analysis of risk factors associated with the probability of survival to hospital discharge. Lastly, it’s important to note that our study focused solely on assessing short-term outcomes in patients. Long-term survival analysis data was not an aim of the current study. Therefore, exploring the relationship between risk factors and predicting long-term outcomes, such as median survival time or the recurrence of CHF, remains an avenue for further investigation.

In conclusion, our study found that the survival probability for dogs with CPE and MMVD was relatively high, with all non-survivors experiencing cardiac-related death during the first 24 h of hospitalization. No clinical parameter measured before initiating treatment was associated with in-hospital mortality. Furthermore, we observed that rehospitalization due to CPE and cardiac death within 2 months after discharge were quite frequent in these dogs. Risk factors such as systolic blood pressure below 90 mmHg upon hospital presentation, administered parenteral inotropes and the minimum respiratory rate during the initial 12 h have been associated with an increased risk of cardiac death within 2 months post-discharge.

## Data Availability

The raw data supporting the conclusions of this article will be made available by the authors, without undue reservation.

## Footnotes

1 CX 50, Philips Ultrasound, Bothell, Washington, equipped with either a 12–4 MHz or an 8–3 MHz phased-array transducer.

2 Vet HDO Monitor S + B medVet GmbH, Babenhausen, Germany.

3 Ultrasonic Doppler Flow Detector Model 811-B, PARKS Medical Electronics, Inc, Aloha, Oregon.

4 ICU-V12, Menix, Tokyo, Japan.

5 MedCalc® Statistical Software version 22.006, MedCalc Software Ltd, Ostend, Belgium; https://www.medcalc.org; 2023.
