# Supplementary material for: Clinical findings and risk factors for clinical outcomes in dogs with myxomatous mitral valve disease hospitalized for cardiogenic pulmonary edema
Source: Front Vet Sci. 2026 May 8;13:1749038. doi: 10.3389/fvets.2026.1749038 (PMC13194064; doi:10.3389/fvets.2026.1749038)
Supplement: Supplementary file 1 [file Table_1.pdf]

Table 1. List of medications administered before presentation to the hospital for onset of acute CPE.

Drugs	Number of Dogs	Median dose (range)(mg/kg/day)
Pimobendan	73	0.7 (0.3-5.1)
Furosemide	42	2.0 (0.2-6.0)
Torsemide	16	0.3 (0.1-1.6)
Spironolactone	49	2.0 (0.9-9.7)
ACEI	32	1.0 (0.2-1.0)
Sildenafil	14	2.1 (1.0-6.6)
Amlodipine	9	0.2 (0.1-0.4)

Abbreviations: ACEI, angiotensin-converting enzyme inhibitors.

Note: ACEI included enalapril and benazepril.
