# Supplementary material for: Clinical findings and risk factors for clinical outcomes in dogs with myxomatous mitral valve disease hospitalized for cardiogenic pulmonary edema
Source: Front Vet Sci. 2026 May 8;13:1749038. doi: 10.3389/fvets.2026.1749038 (PMC13194064; doi:10.3389/fvets.2026.1749038)
Supplement: Supplementary file 2 [file Table_2.pdf]

Table 2. Variables associated with the duration of oxygen administration (hours) identified in the multiple linear regression analysis.

Variable	*P* value	Coef (95% CI)	Std. Error	t	VIF
Total furosemide dose during 12-24 hours (mg/kg)	<.0001	1.1631 (.6857-1.6405)	.2402	4.8426	1.926
Administration of parenteral inotropes (y/n)	<.0001	23.4152 (13.7918-33.0385)	4.8417	4.8362	1.383
Pre-hospitalization dose of furosemide (mg/kg)	.0011	3.7487 (1.5476-5.9498)	1.1074	3.3852	1.012
MMLIS upon follow up	.019	2.5388 (.4273-4.6503)	1.0623	2.3899	2.055

Abbreviations: MMLIS, Modified Murray Lung Injury Score; TDS, Tufts Dyspnea Score. Variables that exhibited positive associations in univariable linear regression analysis were included in the multivariable stepwise selection multiple regression analysis, which identified the following factors: higher cumulative parenteral furosemide dose during the first 12 hours ($P < .001$) and the 12-24 hour period ($P < .001$); higher MMLIS upon follow-up thoracic radiographs ($P < .001$); administration of parenteral inotropes ($P < .001$); longer time required for respiratory rate to drop below 40 breaths per minute ($P = .03$); higher median respiratory rate during the first 12 hours ($P = .04$); elevated TDS at presentation ($P = .02$); and higher minimum ($P < .001$), median ($P < .001$), and maximum ($P = .01$) TDS during the first 12 hours. Additionally, longer time for TDS to drop below 3 ($P = .001$), as well as higher pre-hospitalization doses of furosemide ($P = .01$) and torsemide ($P = .02$) were also noted.

Example of the interpretation: Dogs administered with parenteral inotropes had an estimated 23-hour longer duration of oxygen administration compared to dogs that did not receive inotropes.
