# Supplementary material for: Clinical findings and risk factors for clinical outcomes in dogs with myxomatous mitral valve disease hospitalized for cardiogenic pulmonary edema
Source: Front Vet Sci. 2026 May 8;13:1749038. doi: 10.3389/fvets.2026.1749038 (PMC13194064; doi:10.3389/fvets.2026.1749038)
Supplement: Supplementary file 3 [file Table_3.pdf]

Table 3. Variables associated with survival to hospital discharge identified in the multivariable logistic regression analysis.

Variable	Coef	Std. Error	*P* value	Odds ratio (95% CI)
Minimum TDS during 12 hours	-1.6896	.5685	.003	.18 (.06-.56)

Abbreviations: TDS, Tufts Dyspnea Score.

Variables that exhibited positive associations in the univariable logistic regression analysis were included in the multivariable stepwise selection logistic regression analysis. The following factors were identified: presence of bilateral pulmonary crackles at presentation ($P = .01$), higher minimum ($P < .001$), median ($P = .003$), and maximum ($P = .004$) respiratory rates during the first 12 hours, as well as higher minimum ($P < .001$), median ($P = .003$), and maximum ($P = .01$) TDS during the initial 12 hours.

Example of the interpretation: Each one-unit increase in minimum TDS during the initial 12 hours of hospitalization was associated with an 82% decrease in the odds of a dog surviving to hospital discharge.
