# Supplementary material for: Clinical findings and risk factors for clinical outcomes in dogs with myxomatous mitral valve disease hospitalized for cardiogenic pulmonary edema
Source: Front Vet Sci. 2026 May 8;13:1749038. doi: 10.3389/fvets.2026.1749038 (PMC13194064; doi:10.3389/fvets.2026.1749038)
Supplement: Supplementary file 4 [file Table_4.pdf]

Table 4. Variables associated with cardiac deaths within 2 months post-discharge identified in the multivariable Cox proportional hazards analysis.

**Variable**	**Coef**	**Std. Error**	***P* value**	**Hazards ratio (95% CI)**
SBP <90 mmHg at hospital presentation (y/n)	1.8395	.7928	.02	6.3 (1.3-30)
Administration of parenteral inotropes (y/n)	1.6725	.7434	.025	5.3 (1.2-22)
Minimum RR within 12 hours (bpm)	.0892	.0295	.003	1.1 (1.0-1.2)

Abbreviations: RR, Respiratory rate; bpm, breaths per minutes; SBP, Systolic blood pressure.

Variables that exhibited positive associations in the univariable Cox proportional hazards analysis were included in the multivariable stepwise selection Cox proportional hazards analysis. The following factors were higher cumulative parenteral furosemide dose within the first 24 hours (*P* = .03); higher MMLIS at hospital presentation (*P* = .02) and at follow-up (*P* = .003); administration of parenteral inotropes (*P* = .0008); higher minimum (*P* = .0005) and median (*P* = .01) respiratory rate during the first 12 hours; systolic blood pressure below 90 mmHg at hospital presentation (*P* = .002); longer time for TDS to drop below 3 (*P* = .007); and higher minimum (*P* = .0002), median (*P* = .006), and maximum (P = .01) TDS during the initial 12 hours.

Example of the interpretation: Dogs administered parenteral inotropes had 5.3 times the hazard of cardiac death within 2 months post-discharge compared to dogs that did not receive parenteral inotropes.
