# Supplementary material for: Clinical findings and risk factors for clinical outcomes in dogs with myxomatous mitral valve disease hospitalized for cardiogenic pulmonary edema
Source: Front Vet Sci. 2026 May 8;13:1749038. doi: 10.3389/fvets.2026.1749038 (PMC13194064; doi:10.3389/fvets.2026.1749038)
Supplement: Supplementary file 5 [file Table_5.pdf]

Table 5. Variables associated with rehospitalization due to CPE within 2 months post-discharge identified in the multivariable Cox proportional hazards analysis.

| **Variable** | **Coef** | **Std. Error** | ***P* value** | **Hazard ratio (95% CI)** |
|---|---|---|---|---|
| Grade of murmur | 1.8677 | .4920 | .0001 | 6.5 (2.5-17) |

Variables that exhibited positive associations in the univariable Cox proportional hazards analysis were included in the multivariable stepwise selection Cox proportional hazards analysis. The following factors were higher cumulative parenteral furosemide dose during the 12-24 hour period ($P$ = .05), higher grade of murmur ($P$ = .0001) and higher pre-hospitalization dose of torsemide ($P$ = .01).

Example of the interpretation: Each one-unit increase in the grade of murmur was associated with a 6.5 times increase in the hazard of rehospitalization due to CPE within 2 months post-discharge.
