# Supplementary material for: Clinical findings and risk factors for clinical outcomes in dogs with myxomatous mitral valve disease hospitalized for cardiogenic pulmonary edema
Source: Front Vet Sci. 2026 May 8;13:1749038. doi: 10.3389/fvets.2026.1749038 (PMC13194064; doi:10.3389/fvets.2026.1749038)
Supplement: Supplementary file 6 [file Data_Sheet_1.pdf]

Appendix 1: Tufts Subjective Dyspnea Scale for Dogs and Cats

This scale is intended to categorize the severity of respiratory distress in dogs and cats with cardiopulmonary disease. The dyspnea score should be obtained when the animal is at rest, ideally in a cage or when resting quietly on the floor, following 1 minute of observation of respiratory rate and effort. Cats should not be purring and dogs should not be panting. If dogs are panting or if cats are purring then no score should be recorded. The goal is for you to assess whether you believe an individual dog or cat has the sensation of difficulty breathing, and if so then to score the degree of difficulty breathing. The Criteria to Assess column should be used as a guideline (e.g., many individuals with this score have this parameter), rather than an absolute criteria for scoring. Features to watch besides those listed below might include extended head and neck, nasal flaring, open mouth breathing, cyanosis, or abnormal chest wall or abdominal excursions.

**Score**	**Subjective Assessment**	**Criteria to Assess**
0	No dyspnea	Normal respiratory rate and effort
1		Mild increased respiratory effort, usually with normal respiratory rate (< 30-35 breaths per minute)
2		Mild increase in respiratory rate, respiratory effort or both

3	Mild dyspnea	Increased abdominal effort to breathing is clearly present; animal is still comfortable in either sternal or lateral recumbency
4		
5		More significant difficulty breathing; most animals with score 5 and above might be hypoxemic or be candidates for oxygen administration
6	Moderate dyspnea	Moderate increase in respiratory rate and/or effort; animals may prefer to stay in sternal recumbency or standing; head bob to breathing may be noted
7		
8		Marked to severe respiratory effort; respiratory rate typically > 50-60 breaths per minute
9	Severe dyspnea	Concern for developing respiratory failure, animal may have orthopnea, usually is uncomfortable lying down, unlikely to be in lateral recumbency
10		Respiratory failure imminent or animal is in a near agonal state; mechanical ventilation likely is indicated to prevent cardiopulmonary arrest

Note:

Modified from the Borg Dyspnea Scale

https://pulmonaryrehab.com.au/wp-content/uploads/2022/06/04_modified_borg_dyspnoea_scale.pdf

Scores 4 and 7 on the scale were deliberately left empty as they serve as transitional scores between 3 and 5, and between 6 and 8, respectively. As an illustration, if an animal exhibits characteristics outlined for a score of 3 as well as certain attributes associated with a score of 5, it would be appropriate to assign it a score of 4.
